# The complete mitochondrial genome of an Asian longicorn beetle*, Neoplocaederus obesus* (Gahan) (Coleoptera: Cerambycidae: Cerambycinae)

**DOI:** 10.1080/23802359.2020.1787891

**Published:** 2020-07-06

**Authors:** Shengbo Li, Wen Lu

**Affiliations:** Guangxi Key Laboratory of Agric-Environment and Agric-Products Safety, National Demonstration Center for Experimental Plant Science Education, College of Agriculture, Guangxi University, Nanning, Guangxi, China

**Keywords:** longicorn beetle, *Neoplocaederus obesus*, mitochondrial genome

## Abstract

In this study, the complete mitochondrial genome of the *Neoplocaederus obesus* (GenBank accession number: MT525962) was sequenced. The 15,683 bp long genome has the standard metazoan complement of 38 genes. These genes contain 13 protein-coding genes, 22 transfer RNA genes, 2 ribosomal RNA genes, and 1 control region. The nucleotide composition of the N. obesus mitogenome was A: 37.20%, T: 32.19%, G: 12.10%, C: 18.52%. The A＋T content was 69.39%, showing strong AT skew.

## Introduction

*Neoplocaederus obesus* (Coleoptera: Cerambycidae: Cerambycinae) is an Asian longicorn beetle. It’s considered a vital pest that mainly damages cashew trees (Vasanthi and Raviprasad 2012). *N. obesus* is widely distributed in South and Southeast Asia, such as India, the Andaman Islands (Bulganin et al. 2016), southern China, Thailand, Myanmar, Vietnam, SriLanka and Bangladesh (Bulganin et al. 2016). *N. obesus* larvae damage the cashew trees by tunneling the internal stem base and the vascular tissues of main root (Vasanthi and Raviprasad 2012) and eating under the skin, which finally weaken the tree in the early stage, affect the growth and yield and cause plant death in the later stage. *N. obesus* was found to have only one generation in a year based on the field and laboratory observations (Meshram et al. 2009). These new mitochondrial genome data are more complete and can be better used to provide a basis for studies of the mitochondrial evolution of Cerambycidae and identify larva species (Wang et al. 2019). Here, the complete mitochondrial DNA (mtDNA) genome of *N. obesus* was elucidated which has not been reported before.

In this study, the adult specimens of *N. obesus* were collected on *Dracontomelon duperreanum* Pierre from Guangxi University (22°84′N, 108°29′E), Nanning, Guangxi Province, China. The modified CTAB DNA extraction protocol was used to extract the total genomic DNA. The genomic DNA is available with sample number of (SZHT0603G055-2) and stored at Guangxi Key Laboratory of Agric-Environment and Agric-Products Safety (Nanning, China). Subsequently, the library preparations were sequenced on an Illumina HiSeq platform and 2 × 150 bp paired-end reads were generated. Approximately 11.39 G of raw data and 11.14 G of clean data were obtained for sequence assembly by using virtue of SPAdes (version 3.11.1) (Bankevich et al. 2012).

The complete mitochondrial genome of *N. obesus* is a closed circular molecule (GenBank accession number MT525962), which composed of 38 genes. The mitogenome was submitted to NCBI GenBank. The genome comprised 38 genes, including 13 protein-coding genes (PCGs), 22 transfer RNA (tRNA) genes, 2 ribosomal RNA (rRNA) genes, and 1 control region. The length of the complete mitochondrial genome of *N. obesus* is 15,683bp, with an A + T content of 69.39% and the following base composition: A (37.20%), T (32.19%), G (12.10%), C (18.52%). The composition of the genome sequence is highly asymmetric with an obvious bias toward A and T.

**Figure 1.**
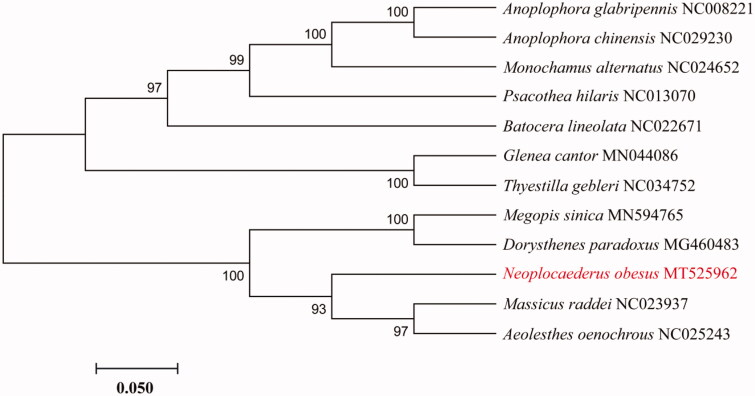
Neighbor-joining phylogenetic tree of *Neoplocaederus obesus* and other Prioninae, Cerambycinae and Lamiinae beetles. The complete mitochondrial genome was downloaded from GenBank and the phylogenic tree was constructed by Neighbor-Joining method with 1000 bootstrap replicates. MtDNA accession numbers used for tree construction are as follows: *Anoplophora chinensis* (NC029230), *Anoplophora glabripennis* (NC008221), *Monochamus alternatus* (NC024652), *Psacothea hilaris* (NC013070), *Batocera lineolata* (NC022671), *Thyestilla gebleri* (NC034752), *Glenea cantor* (MN044086), *Dorysthenes paradoxus* (MG460483), *Aeolesthes oenochrous* (NC025243), *Massicus raddei* (NC023937).

Molecular Evolutionary Genetics Analysis Version 7.0 (MEGA7.0) was used to construct the phylogenetic tree with the neighbour-joining method among some species from Prioninae, Cerambycinae and Lamiinaerespectively. Branch support was estimated by boot strap analysis 1000 replicates (Sudhir et al. 2016). Phylogenetic analysis showed that *N. obesus* is closely related to *Aeolesthes oenochrous* and *Massicus raddei* (Figure 1).

## Data Availability

The data that support the findings of this study are openly available in GenBank BankIt in NCBI at [https://www.ncbi.nlm.nih.gov/], reference number MT525962. Data is ethically correct to do and does not violate the protection of human subjects, or other valid ethical, privacy, or security concerns.
